# When the Body Falls Silent: A Case Report of Amyotrophic Lateral Sclerosis

**DOI:** 10.7759/cureus.95427

**Published:** 2025-10-26

**Authors:** Marta Pereira de Oliveira, Filomena Lima Monteiro

**Affiliations:** 1 Family Medicine, USF Ars Médica, Lisboa, PRT

**Keywords:** amyotrophic lateral sclerosis, early-onset als, motor neuron, multidisciplinary care, neurodegenerative disorder, neuromuscular symptoms, palliative medicine, respiratory failure

## Abstract

Amyotrophic lateral sclerosis (ALS) is a progressive neurodegenerative disorder affecting both upper and lower motor neurons, resulting in muscle weakness, atrophy, and, ultimately, respiratory failure.

We present the case of a 49-year-old man who sustained a right wrist injury in January 2023, for which he sought evaluation by his general practitioner (GP). Due to an unfavorable recovery, he was referred to the emergency department (ED) in September of the same year, where the diagnostic workup was initiated. Following a series of complementary diagnostic tests, a diagnosis of ALS was established at the end of September. Electromyography demonstrated widespread denervation with both acute and chronic neurogenic changes, while complementary diagnostic studies excluded alternative etiologies. The patient subsequently began follow-up with a multidisciplinary team encompassing the various domains of ALS management over the following 17 months. Riluzole therapy was initiated. He ultimately passed away in February 2025, having demonstrated remarkable resilience throughout the course of his illness.

This case highlights the devastating impact of early-onset ALS and underscores the importance of maintaining clinical suspicion when evaluating adults presenting with progressive neuromuscular symptoms. The rapid disease progression and associated psychosocial burden emphasize the critical role of multidisciplinary management, early palliative integration, and strong primary care involvement in optimizing patient and family support.

## Introduction

Amyotrophic lateral sclerosis (ALS) is a progressive neurodegenerative disease of the motor neurons, characterized by signs of both upper and lower motor neuron involvement, leading to weakness, muscle atrophy, and, in advanced stages, respiratory failure. The estimated incidence is 0.7 to 3.8 per 100,000 people per year, and the prevalence ranges between 4 to 10 per 100,000, with a peak onset between 60 and 79 years of age[1,2]. Nevertheless, a subset of cases occurs at younger ages. Juvenile ALS, defined by onset before age 25, is rare and often associated with monogenic mutations. Cases between 25 and 45 years are typically classified as early-onset ALS and may, in some cases, present a genetic predisposition without a clear Mendelian pattern [3,4].

Although ALS management remains largely focused on supportive care and multidisciplinary interventions, recent therapeutic advances, including gene-targeted therapies such as tofersen for SOD1-related ALS and other emerging disease-modifying approaches, illustrate how treatment strategies are beginning to evolve, particularly for patients with specific genetic subtypes [5].

Case reports remain essential to better understanding the clinical and genetic heterogeneity of ALS, as well as to emphasizing the importance of early diagnosis and integration of multidisciplinary care. This report describes an early-onset ALS case with rapid progression, highlighting clinical, social, and emotional challenges.

## Case presentation

This case involved a 49-year-old Caucasian male, married and employed as a warehouse worker, who lived with his wife and 13-year-old daughter on the fourth floor of a building without an elevator. He had a smoking habit and a history of right upper limb trauma 30 years earlier.

In April 2023, he consulted his general practitioner (GP) with complaints of right wrist pain following trauma sustained three months earlier. An ultrasound revealed tenosynovitis of the fourth extensor compartment, and he was referred to Physical Medicine and Rehabilitation (PMR).

On September 6, 2023, during a PMR consultation, he was referred to the Emergency Department (ED) at Hospital Beatriz Ângelo (HBA) due to progressive weakness in the right upper limb (RUL). He reported difficulties performing tasks such as writing or handling a knife. These were associated with fasciculations and functional limitation of the right lower limb (RLL), affecting gait.

Neurological examination in the ED revealed flaccid monoplegia with areflexia and marked atrophy of the shoulder girdle and RUL with fasciculations, without sensory changes. He was hemodynamically stable, with no other significant findings on neurological examination. A brain CT was unremarkable. He was electively admitted to the neurology ward at HBA from September 11 to 29, 2023, for further study, with differential diagnoses of motor neuron disease versus cervical myelopathy. Complementary diagnostic tests performed are summarized in Tables 1-2.

**Table 1:** Diagnostic and laboratory findings (2023). CBC, complete blood count; ESR, erythrocyte sedimentation rate; CRP, C-reactive protein; PTH, parathyroid hormone; RPR, rapid plasma reagin; HIV, human immunodeficiency virus; HBV, hepatitis B virus; HCV, hepatitis C virus; ACE, angiotensin-converting enzyme; PSA, prostate-specific antigen; IgA, immunoglobulin A; ANA, antinuclear antibody; anti-dsDNA, anti-double-stranded DNA antibody; ANCA, antineutrophil cytoplasmic antibody; RF, rheumatoid factor; CCP, cyclic citrullinated peptide; SSA/SSB, Sjögren’s-syndrome-related antigen A and B; EMG, electromyography; CSF, cerebrospinal fluid; CT, computed tomography

Diagnostic test	Results	Normal range/expected finding
Brain CT (September 06, 2023)	No significant focal densitometric abnormalities. Ventricular and cisternal systems patent. Cortical sulci preserved. No extra-axial fluid or hemorrhagic collections.	Normal brain CT: No focal lesions, normal ventricular and cisternal configuration, preserved cortical sulci, and absence of hemorrhage or extra-axial collections.
Cervical CT (September 13, 2023)	Mild posterior disco-osteofibrotic ridges between C3 and C5, slightly obliterating the anterior perimedullary CSF space. Relative left C4–C5 foraminal stenosis, possibly affecting the left C5 root.	Normal cervical CT: Preserved vertebral alignment, intact disc spaces, and no canal or foraminal stenosis.
EMG (September 12, 2023)	Electromyographic signs of motor neurogenic dysfunction in cervical and lumbosacral regions, with active denervation.	Normal EMG: No evidence of active denervation or neurogenic changes.
Laboratory tests (09/2023)	Unremarkable CBC; ESR 21 mm/hour; CRP 0.5 mg/dL; glucose 91 mg/dL; normal renal, liver, and thyroid function; PTH 22.5 pg/mL; negative RPR, HIV, HBV, HCV; no folate or B12 deficiency; ACE 71 U/L; PSA 0.59 ng/mL; normal protein electrophoresis; IgA slightly elevated 6.35 g/L; negative autoimmunity (ANA, anti-dsDNA, ANCA, RF, CCP, SSA/SSB). 24-hour urine: lead and Bence-Jones proteins negative.	Normal ranges: ESR <20 mm/hour; CRP <1 mg/dL; glucose 70-99 mg/dL; PTH 15-65 pg/mL; ACE 8-52 U/L; PSA <4 ng/mL; IgA 0.7-4.0 g/L; normal renal, hepatic, and thyroid profiles; negative infectious and autoimmune markers; normal urine without Bence-Jones proteins or heavy metals.

**Table 2:** Cerebrospinal fluid (CSF) findings.

Parameter	Result	Normal range/expected finding
Appearance	Hemorrhagic	Normally clear and colorless
WBC	4 cells/μL	<5 cells/μL
Glucose	67.2 mg/dL	50-80 mg/dL
Protein	54.6 mg/dL	15-45 mg/dL
LDH	16 U/L	<40 U/L
Lactate	1.5 mmol/L	<2.1 mmol/L

He was discharged with a definitive diagnosis of ALS, prescribed riluzole and baclofen, and referred to pulmonology, PMR, and neurology consultations.

In the initial weeks of treatment, he reported partial improvement in lower limb motor symptoms, though fasciculations and spontaneous clonus persisted. On December 12, 2023, he underwent botulinum toxin treatment.

Over subsequent weeks, progressive dependence in mobility developed. Multidisciplinary consultations optimized supportive care. He and his family also received support from the Portuguese ALS Association (APELA) for guidance regarding social benefits and rights.

By January 2024, he was partially dependent in activities of daily living (ADLs), using a wheelchair for mobility. Symptomatically, he developed dysphagia for liquids and dyspnea on moderate exertion. Physical examination showed marked muscle atrophy, particularly in the upper limbs, generalized fasciculations, generalized hyperreflexia, and axial motor deficit. Pulmonary function tests led to the recommendation of non-invasive ventilation, which he had difficulty adapting to.

The following months were marked by rapid deterioration, with further loss of upper limb functionality, worsening dysphagia, muscle spasms, and fatigue. By March 2024, he was fully dependent for ADLs. In April 2024, he underwent percutaneous endoscopic gastrostomy (PEG), following evaluation by nutrition and ENT teams. He continued limited oral intake of soft foods by personal preference despite the PEG. In May 2024, analgesic and anxiolytic therapy was adjusted, initiating morphine for pain control.

He was referred to the Integrated Palliative Care Team of Loures for home-based support. Over the months, the psychological impact became evident, with episodes of anxiety and panic, refusal of therapies, and resistance to assistive devices. The family had to significantly adjust their lives, including work, to accompany him throughout the disease course. The home was progressively adapted with an articulated bed, a visually controlled television, among other assistive aids.

Weekly follow-ups were conducted by the multidisciplinary team and palliative care providers. By October 2024, he increasingly refused to leave home or attend physiotherapy sessions at APELA. During the last semester of 2024, his overall condition deteriorated significantly. He was hospitalized in January 2025 with community-acquired pneumonia and passed away the following month.

## Discussion

This case illustrates the devastating nature of ALS, particularly when it affects individuals in the most active phase of their lives. The atypical initial presentation, initially attributed to an orthopedic injury, delayed the recognition of an underlying neuromuscular disorder, a common diagnostic challenge in early-onset ALS. Early use of electromyography, patient education, and awareness of red-flag symptoms could help reduce such delays and facilitate timely diagnosis [1,3].

The rapid clinical progression, difficulty adapting to physical limitations, and psychological distress experienced by both the patient and family underscore the urgent need for integrated, multidisciplinary care across all levels of the healthcare system [4]. The family physician played a pivotal role in ensuring timely referral, coordinating specialized care, and mobilizing family and community support networks, consistent with findings that highlight the survival and quality-of-life benefits of multidisciplinary management [4].

Despite available therapeutic options, current resources remain insufficient to achieve favorable outcomes in ALS. This case reinforces the importance of maintaining a high index of suspicion for ALS in adults presenting with progressive neuromuscular symptoms [1,2], adopting a patient-centered approach, and integrating palliative care early in the disease course [4].

## Conclusions

This early-onset ALS case highlights the importance of maintaining a high index of suspicion when evaluating adults with progressive neuromuscular symptoms.

The rapid disease progression, coupled with profound social and psychological challenges, emphasizes the need for multidisciplinary management and the early integration of palliative care. Family physicians play a critical role in facilitating timely referrals, coordinating comprehensive care, and supporting patients and families as they navigate the complex course of ALS. Further research and continued reporting of similar cases are essential to improve early recognition and enhance multidisciplinary management of this devastating disease.
